# Supplementary material for: Multi‐dataset identification of innovative feature genes and molecular mechanisms in keratoconus
Source: J Cell Mol Med. 2024 Sep 19;28(18):e70079. doi: 10.1111/jcmm.70079 (PMC11412914; doi:10.1111/jcmm.70079)
Supplement: Supplementary file 2 — Material S2. [file JCMM-28-e70079-s002.zip › 7.GSEA analysis/5.RNF217-AS1/KEGG_AUTOIMMUNE_THYROID_DISEASE.html]

Details for gene set KEGG\_AUTOIMMUNE\_THYROID\_DISEASE[GSEA]

|  || Dataset | RNF217-AS1.group.cls#h\_versus\_l |
| Phenotype | group.cls#h\_versus\_l |
| Upregulated in class | h |
| GeneSet | KEGG\_AUTOIMMUNE\_THYROID\_DISEASE |
| Enrichment Score (ES) | 0.31336161 |
| Normalized Enrichment Score (NES) | 0.8582548 |
| Nominal p-value | 0.6633663 |
| FDR q-value | 1.0 |
| FWER p-Value | 1.0 |
Table: GSEA Results Summary

  

Fig 1: Enrichment plot: KEGG\_AUTOIMMUNE\_THYROID\_DISEASE      
 Profile of the Running ES Score & Positions of GeneSet Members on the Rank Ordered List

  

| SYMBOL | TITLE | RANK IN GENE LIST | RANK METRIC SCORE | RUNNING ES | CORE ENRICHMENT || 1 | TSHR | na | 64 | 0.807 | 0.0905 | Yes |
| 2 | CD86 | na | 733 | 0.481 | 0.1263 | Yes |
| 3 | IFNA14 | na | 927 | 0.451 | 0.1723 | Yes |
| 4 | IFNA4 | na | 1517 | 0.391 | 0.2001 | Yes |
| 5 | IFNA16 | na | 1944 | 0.354 | 0.2284 | Yes |
| 6 | IL2 | na | 2070 | 0.345 | 0.2642 | Yes |
| 7 | IFNA6 | na | 2619 | 0.310 | 0.2839 | Yes |
| 8 | CGA | na | 2795 | 0.300 | 0.3132 | Yes |
| 9 | PRF1 | na | 3819 | 0.259 | 0.3134 | Yes |
| 10 | FASLG | na | 8750 | 0.111 | 0.1839 | No |
| 11 | IFNA7 | na | 9097 | 0.103 | 0.1857 | No |
| 12 | CD28 | na | 9337 | 0.098 | 0.1901 | No |
| 13 | TSHB | na | 10024 | 0.086 | 0.1802 | No |
| 14 | CD80 | na | 10316 | 0.081 | 0.1810 | No |
| 15 | IFNA21 | na | 11545 | 0.062 | 0.1527 | No |
| 16 | HLA-DOA | na | 11639 | 0.061 | 0.1570 | No |
| 17 | IFNA2 | na | 12465 | 0.049 | 0.1388 | No |
| 18 | HLA-DMB | na | 13802 | 0.033 | 0.1040 | No |
| 19 | HLA-F | na | 13970 | 0.031 | 0.1028 | No |
| 20 | HLA-DMA | na | 14233 | 0.028 | 0.0984 | No |
| 21 | TPO | na | 15177 | 0.017 | 0.0732 | No |
| 22 | HLA-E | na | 15330 | 0.016 | 0.0706 | No |
| 23 | HLA-A | na | 15413 | 0.015 | 0.0700 | No |
| 24 | HLA-B | na | 17543 | -0.007 | 0.0093 | No |
| 25 | HLA-G | na | 18423 | -0.015 | -0.0143 | No |
| 26 | HLA-C | na | 18927 | -0.019 | -0.0266 | No |
| 27 | HLA-DRB5 | na | 19471 | -0.025 | -0.0394 | No |
| 28 | HLA-DRB1 | na | 23407 | -0.067 | -0.1452 | No |
| 29 | IL5 | na | 23582 | -0.070 | -0.1422 | No |
| 30 | CD40 | na | 23601 | -0.070 | -0.1348 | No |
| 31 | HLA-DRB3 | na | 23619 | -0.070 | -0.1273 | No |
| 32 | GZMB | na | 23756 | -0.072 | -0.1230 | No |
| 33 | TG | na | 24070 | -0.076 | -0.1233 | No |
| 34 | HLA-DOB | na | 26792 | -0.122 | -0.1878 | No |
| 35 | FAS | na | 28081 | -0.151 | -0.2077 | No |
| 36 | CTLA4 | na | 28101 | -0.151 | -0.1909 | No |
| 37 | IL10 | na | 28464 | -0.161 | -0.1830 | No |
| 38 | IFNA8 | na | 29826 | -0.201 | -0.1992 | No |
| 39 | CD40LG | na | 30110 | -0.210 | -0.1833 | No |
| 40 | HLA-DRB4 | na | 30111 | -0.210 | -0.1593 | No |
| 41 | HLA-DPA1 | na | 30869 | -0.237 | -0.1540 | No |
| 42 | HLA-DQB1 | na | 31211 | -0.250 | -0.1352 | No |
| 43 | HLA-DRA | na | 31261 | -0.252 | -0.1077 | No |
| 44 | HLA-DQA1 | na | 32266 | -0.307 | -0.1015 | No |
| 45 | IFNA10 | na | 32398 | -0.317 | -0.0690 | No |
| 46 | IL4 | na | 33228 | -0.378 | -0.0496 | No |
| 47 | HLA-DQA2 | na | 33332 | -0.388 | -0.0081 | No |
| 48 | HLA-DPB1 | na | 33623 | -0.423 | 0.0319 | No |
Table: GSEA details [plain text format]

  

Fig 2: KEGG\_AUTOIMMUNE\_THYROID\_DISEASE      
 Blue-Pink O' Gram in the Space of the Analyzed GeneSet

  

Fig 3: KEGG\_AUTOIMMUNE\_THYROID\_DISEASE: Random ES distribution      
 Gene set null distribution of ES for **KEGG\_AUTOIMMUNE\_THYROID\_DISEASE**
